# Purification and Visualization of Influenza A Viral Ribonucleoprotein Complexes

**DOI:** 10.3791/1105

**Published:** 2009-02-09

**Authors:** Winco W.H. Wu, Lindsay L. Weaver, Nelly Panté

**Affiliations:** Department of Zoology, University of British Columbia - UBC

## Abstract

The influenza A viral genome consists of eight negative-sense, single stranded RNA molecules, individually packed with multiple copies of the influenza A nucleoprotein (NP) into viral ribonulceoprotein particles (vRNPs). The influenza vRNPs are enclosed within the viral envelope. During cell entry, however, these vRNP complexes are released into the cytoplasm, where they gain access to the host nuclear transport machinery. In order to study the nuclear import of influenza vRNPs and the replication of the influenza genome, it is useful to work with isolated vRNPs so that other components of the virus do not interfere with these processes. Here, we describe a procedure to purify these vRNPs from the influenza A virus. The procedure starts with the disruption of the influenza A virion with detergents in order to release the vRNP complexes from the enveloped virion. The vRNPs are then separated from the other components of the influenza A virion on a 33-70% discontinuous glycerol gradient by velocity sedimentation. The fractions obtained from the glycerol gradient are then analyzed on via SDS-PAGE after staining with Coomassie blue. The peak fractions containing NP are then pooled together and concentrated by centrifugation. After concentration, the integrity of the vRNPs is verified by visualization of the vRNPs by transmission electron microscopy after negative staining. The glycerol gradient purification is a modification of that from Kemler *et al.* (1994)^1^, and the negative staining has been performed by Wu *et al.* (2007).^2^

**Figure:**
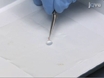


## Protocol

### Part 1: Disruption of the Influenza A Virion

1. Add 750 µl of MNT buffer (20 mM MES, 150 mM NaCl, 30 mM Tris, pH 7.5) into one Beckman polycarbonate centrifuge tube (11 mm x 34 mm) designed to fit into a TLA-120.2 rotor for use in a Beckman Optima Max-E ultracentrifuge.
2. Add 500 µl of the influenza A virus (H3N2 X-31 A/AICHI/68 strain; 2 mg/ml) into that tube. Mix the virus with the MNT buffer by pipetting up and down several times.
3. Centrifuge for 10 minutes at 109,000 x g, 4 °C, in a Beckman Optima Max-E centrifuge using a TLA-120.2 rotor.
4. Remove the supernatant, and resuspend the pellet in 500 µl disruption buffer (100 mM KCl, 5 mM MgCl_2_, 5% (w/v) glycerol, 50 mM octylglucoside, 10 mg/ml lysolecithin, 1.5 mM dithiothreitol, 100 mM MES, pH 5.5).
5. Vortex vigorously, and then shake at 31 °C for 20 minutes in an eppendorf thermomixer.

### Part 2: Glycerol Gradient Sediment Velocity Centrifugation

1. Prepare a glycerol gradient by placing the following amounts of glycerol into a Beckman ultraclear centrifuge tube (13 mm x 51 mm): 1 ml 70% (v/v) glycerol, 0.75 ml 50% glycerol, 0.375 ml 40% glycerol, and 1.8 ml 33% glycerol. Glycerol solutions are made by mixing pure glycerol with NM buffer (150 mM NaCl, 50 mM MES, pH 5.5). Also prepare a balance tube containing both glycerol and buffer.
2. Vortex the disrupted viral sample again, and load it onto the glycerol gradient.
3. Centrifuge the gradient for 3.75 hours at 217,000 x g, 4 °C, in a Beckman MLS-50 swinging-bucket rotor.
4. After the centrifugation is complete, manually collect 250 µl aliquots of the gradient starting from the top of the tube. Keep fractions on ice or at 4 °C.

### Part 3: Analysis of the Glycerol Gradient Fractions by SDS Polyacrylamide Gel Electrophoresis

1. Remove 20 µl from each fraction to a fresh tube.
2. Add 5 µl of 5x SDS-PAGE sample buffer.
3. Heat to 95 °C for 5 minutes.
4. Spin down the samples briefly and load them onto a 10% polyacrylamide gel, and include  molecular weight markers in one of the wells.
5. Run the samples on the gel until the bromphenol blue loading dye reaches close to the bottom of the gel.
6. Stain the gel with Coomassie blue.

### Part 4: Concentration of the vRNP Fractions

1. Choose the glycerol fractions containing mainly NP, combine them, and distribute them into two Beckman polycarbonate centrifuge tubes (11 mm x 34 mm).
2. Fill each tube with diethyl pyrocarbonate (DEPC)-treated Ultrapure water, and pipet up and down several times to mix.
3. Centrifuge for 4.5 hours at 157,000 x g, 4°C, in a Beckman TLA-120.2 rotor.
4. Remove the supernatant, and resuspend the pellet in 50 µl of DEPC-treated water.
5. If desired, the A_280_ of the concentrated vRNPs can be measured. As NP is the major component present in the pooled fractions, an estimate of the molarity of NP can be calculated using its extinction coefficient of 55,350 M^-1^ cm^-1^ (as determined via ProtParam^3^).
6. Aliquot the purified vRNPs, and store them frozen at -80 °C for later use.

### Part 5: Negative Staining of vRNPs

1. Dilute 1 µl of purified vRNP into 9 µl of freshly-made and filtered-twice MNT buffer (20 mM MES, 150 mM NaCl, 30 mM Tris, pH 7.5). Other buffers are also OK to dilute the vRNPs and to wash the specimen grid (step 5.5), but do not use PBS if uranyl acetate is used for negative staining.
2. Glow-discharge a specimen (copper TEM) grid previously coated with a parlodion and carbon film for 30 seconds.
3. Hold the freshly glow-discharged specimen grid with tweezers, and apply a 5-µl drop of diluted vRNP onto the specimen grid.
4. Leave the drop of specimen on the grid for 8 minutes.
5. While waiting, place a small strip of Parafilm on the bench, and dispense 2 drops containing 10 µl each of MNT buffer (to wash the grid), and a 80-µl drop of the freshly made staining solution (1% ammonium molybdate or 1% uranyl acetate) onto the Parafilm.
6. After the 8-minutes of adsorption, wick off part of the sample solution from the specimen grid with a piece of filter paper (cut into triangles). Do not let the specimen grid to dry.
7. Wash specimen grid in 2 drops containing 10 µl each of MNT buffer for a total time of 1 minute. This is done by carefully lowering the specimen grid on the drop, and then wicking off part of the sample solution from the specimen grid with a piece of filter paper (without letting the specimen grid dry).
8. Immediately after wicking off the last drop of buffer from specimen grid, completely submerge the specimen grid within the stain droplet and wait 1 minute.
9. Completely wick off the stain solution from the specimen grid with a piece of filter paper.
10. Allow the specimen grid to air-dry for several minutes before observation under a transmission electron microscope.

### Part 6: Representative Results:


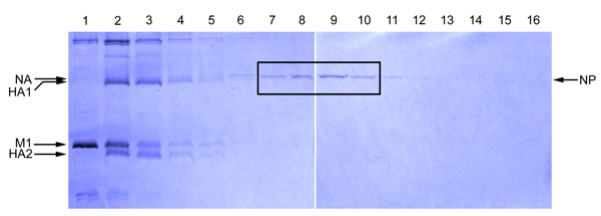


#### Figure 1

As influenza A NP (~56 kDa) is the major protein found in viral ribonucleoprotein complexes, the NP band generally is the strongest band in the fractions containing the vRNPs (Figure 1). In addition to NP, each influenza vRNP also contains a copy of a trimeric RNA polymerase (molecular weights of 82, 86 and 86.5 kDa) complex. These may or may not be visible by Coomassie blue staining because their abundance is low compared to that of NP. The polymerases, however, can be detected if, instead of a Commassie blue stain, a silver stain of the gel is performed. The influenza matrix protein M1 (~ 28 kDa) should be minimally present in the fractions where the NP protein peak fractions are present.


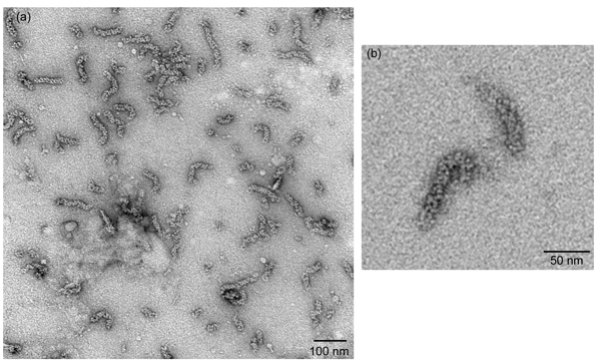


#### Figure 2

Negatively-stained vRNPs as visualized under a transmission electron microscopy should yield vRNPs that resemble rod-shaped particles with variable length that are approximately 30 nm to 120 nm in length (Figure 2a). The oligomeric NP is organized as a chain of NP molecules that is further folded into a double helical repeat structure, so loops on either ends of these rod-shaped particles can sometimes be seen (Figure 2b).

## Discussion

The purification of vRNPs is based on the procedure described by Kemler *et al.* (1994).^1^ We and others have also used this protocol to isolate vRNPs to study their nuclear import.^2,4,5^

We recommend the use of RNase-free tips and tubes when manipulating vRNPs because the viral genome is composed of RNA, and therefore degrades easily in the presence of RNA. In addition, all buffers should be made in water that is RNase free. The protocol described here was performed with an acidic extraction (e.g. the pH of the disruption buffer and the glycerol gradient is 5.5). Similarly, a basic extraction can be performed (as described by Kemler *et al.* (1994)^1^) by substituting MES, pH 5.5 with Tris, pH 7.8.
